# Tolerability to romidepsin in patients with relapsed/refractory T-cell lymphoma

**DOI:** 10.1186/2050-7771-2-16

**Published:** 2014-09-08

**Authors:** Francine Foss, Bertrand Coiffier, Steven Horwitz, Barbara Pro, H Miles Prince, Lubomir Sokol, Matthew Greenwood, Adam Lerner, Dolores Caballero, Eugeniusz Baran, Ellen Kim, Jean Nichols, Barbara Balser, Julie Wolfson, Sean Whittaker

**Affiliations:** 1 Yale Cancer Center, PO Box 208028, 333 Cedar Street, TMP 3, New Haven, CT 06520-8028, USA; 2 Hospices Civils de Lyon, Lyon, France; 3 Memorial Sloan Kettering Cancer Center, New York, NY, USA; 4 Thomas Jefferson University, Philadelphia, PA, USA; 5 Peter MacCallum Cancer Centre and University of Melbourne, East Melbourne, Victoria, Australia; 6 Moffitt Cancer Center, Tampa, FL, USA; 7 Royal North Shore Hospital, Sydney, New South Wales, Australia; 8 Boston Medical Center, Boston, MA, USA; 9 Hospital Universitario de Salamanca, Salamanca, Spain; 10 Wroclaw Medical University, Wroclaw, Poland; 11 University of Pennsylvania, Philadelphia, PA, USA; 12 J Nichols LLC, Swampscott, MA, USA; 13 Veristat, LLC, Holliston, MA, USA; 14 Guy’s and St Thomas’ Hospital, London, England, United Kingdom

**Keywords:** Romidepsin, CTCL, PTCL, Adverse events, Discontinuations

## Abstract

**Background:**

Histone deacetylase inhibitor romidepsin has demonstrated durable clinical responses and tolerability in patients with relapsed/refractory peripheral and cutaneous T-cell lymphoma (PTCL, CTCL). Selection of novel drug therapies for patients with relapsed/refractory aggressive lymphoma requires not only considerations regarding efficacy but also careful evaluation of toxicities as well as overall clinical benefit. The purpose of this analysis was to examine common adverse events (AEs) reported in pivotal trials of romidepsin in relapsed/refractory PTCL or CTCL and to more clearly define the overall AE profile in these populations.

**Methods:**

Patients with relapsed/refractory PTCL or CTCL were treated with romidepsin at 14 mg/m^2^ as a 4-hour intravenous infusion on days 1, 8, and 15 of 28-day cycles for up to 6 cycles; patients with at least stable disease could extend therapy until progressive disease or another withdrawal criterion was met. All enrolled patients who received ≥ 1 dose of romidepsin were included in the AE analyses.

**Results:**

Overall, safety profiles of common AEs were similar, although patients with relapsed/refractory PTCL had more frequent hematologic toxicities and grade ≥ 3 infections. In both patient populations, the greatest incidence of grade ≥ 3 AEs and the majority of discontinuations due to AEs occurred during cycles 1–2. Early discontinuations were primarily related to infection, thrombocytopenia, or electrocardiogram abnormalities, confirming the need to closely monitor patients with poor bone marrow reserve or other comorbidities. Despite this, 28% of patients with relapsed/refractory PTCL and 36% of patients with relapsed/refractory CTCL continued on romidepsin treatment for ≥ 6 cycles.

**Conclusions:**

This study demonstrates that patients with relapsed/refractory PTCL or CTCL have similar AE profiles with romidepsin treatment, although patients with PTCL experienced more frequent and more severe hematologic toxicities and more frequent grade ≥ 3 infections. The greatest incidence of grade ≥ 3 AEs and the majority of discontinuations due to AEs occurred during treatment cycles 1–2. Extended dosing of romidepsin can be tolerated in responding patients.

**Trial registration:**

NCT00426764,NCT00106431

## Background

Romidepsin—a structurally unique, potent, bicyclic class 1 selective histone deacetylase inhibitor [1-3]—is approved by the United States Food and Drug Administration for patients with cutaneous T-cell lymphoma (CTCL) who have received at least one prior systemic therapy and patients with peripheral T-cell lymphoma (PTCL) who have received at least one prior therapy [4].

CTCL is a primarily indolent, heterogeneous group of non-Hodgkin lymphoma (NHL) with a poor prognosis in advanced stage disease [5]. CTCL arises when CD4+ malignant T cells localize to the skin [6]; however, in later disease stages, patients may also have lymph node, blood, and/or visceral involvement [7]. Patients with CTCL often experience intolerable itching (pruritus), visual (cosmetic) skin changes, and frequent infections [8-10]. PTCL is an aggressive, uncommon form of NHL typically associated with a poor prognosis [11]. Disease arises from mature, post-thymic T cells or natural killer (NK) cells [12]. Clinical features vary widely in this heterogeneous group of diseases, with varying symptoms and organ involvement. However, hematologic abnormalities are common in patients with PTCL and may be due to disease involvement in the bone marrow or prior myelosuppressive chemotherapy [13]. Durable clinical responses in PTCL or advanced-stage CTCL are difficult to achieve [5,12,14].

A phase 1 trial conducted by the National Cancer Institute (NCI) demonstrated activity of romidepsin in T-cell lymphoma [15]. A phase 2 NCI trial was then initiated to evaluate the safety and efficacy of romidepsin in relapsed or refractory (R/R) CTCL or PTCL [16,17]. Based on initial results from the NCI trial, separate pivotal registration trials were also conducted in each indication: GPI-04-0001 in R/R CTCL [18] and GPI-06-0002 in R/R PTCL [13].

In GPI-04-0001, single-agent romidepsin therapy resulted in durable responses in patients with R/R CTCL who had received at least one prior systemic therapy with an objective response rate (ORR) of 34% (33/96, including 6% [6/96] complete response [CR]) and median duration of response (DOR) of 15 months (range, < 1-20+; median follow-up not reported) [18]. Similar responses to romidepsin were observed in all stages of disease and across all disease compartments: skin, lymph nodes, and blood [18]. The most common romidepsin-related adverse events (AEs) in CTCL were gastrointestinal or asthenic conditions, primarily grade 1–2 [18].

In GPI-06-0002, patients with R/R PTCL achieved durable responses with romidepsin treatment, with an ORR of 25% (33/130, including 15% [19/130] confirmed/unconfirmed CR) [13] and median DOR of 28 months (range < 1-48+) with median follow-up of 22.3 months [19]. Romidepsin demonstrated comparable efficacy in the 3 most common PTCL subtypes: PTCL not otherwise specified, angioimmunoblastic T-cell lymphoma, and anaplastic lymphoma kinase–negative anaplastic large cell lymphoma. Patient baseline characteristics or prior treatments did not affect the response [13]. The most frequent romidepsin-related AEs were nausea and asthenia/fatigue, which were primarily grade 1–2 and did not result in drug discontinuation [13].

In both R/R CTCL and R/R PTCL, romidepsin was not correlated with clinically meaningful QTc prolongation [13,18,20], and a similar toxicity profile was observed [4,13,18]. The purpose of this analysis was to examine common AE data in detail from the pivotal trials of romidepsin in R/R CTCL and R/R PTCL and to evaluate the AE profile in these 2 groups of patients.

## Results

### Patient characteristics

Baseline characteristics in R/R CTCL or R/R PTCL were similar and were previously described [13,18]. Notably, patients with R/R CTCL or R/R PTCL in these trials tended to be heavily pretreated (median of 2 [PTCL] or 3 [CTCL] prior systemic therapies [range, 1–8 in both]), and > 70% of patients in each trial had advanced disease (III-IV for PTCL, IIB-IVA for CTCL). The majority of patients received prior chemotherapy, with fewer patients receiving prior monoclonal antibodies, immunotherapy, or novel agents. All patients with R/R CTCL had previously received at least one topical therapy [13,18].

### Romidepsin exposure

The median durations of treatment were 1.4 months (range, < 0.1-35.7 months) and 3.5 months (<0.1-26.7 months) for patients with PTCL or CTCL, respectively. There was a significant population of early withdrawals during cycles 1–2: 72 (55%) and 28 (27%) patients with PTCL or CTCL, respectively. For patients with PTCL, these early withdrawals were due to disease progression (n = 50), adverse events (n = 16), withdrawal of informed consent (n = 2), or other reasons (n = 4). For patients with CTCL, these early withdrawals were due to disease progression (n = 10), adverse events (n = 11), withdrawal of informed consent (n = 6), or other reasons (n = 1). Although the AEs leading to early discontinuation varied widely in both patient populations, they were primarily related to infections, thrombocytopenia, or ECG abnormalities. Early discontinuation due to hematologic AEs was uncommon in both patient populations. Many patients tolerated romidepsin for at least 6 cycles: 36 (28%) and 36 (36%) patients with PTCL or CTCL, respectively. Patients received routine antiemetic prophylaxis prior to each romidepsin dose. The most common antiemetics administered were 5HT3 antagonists ondansetron and granisetron.

### Incidence of common AEs in PTCL or CTCL populations

**Figure 1:**
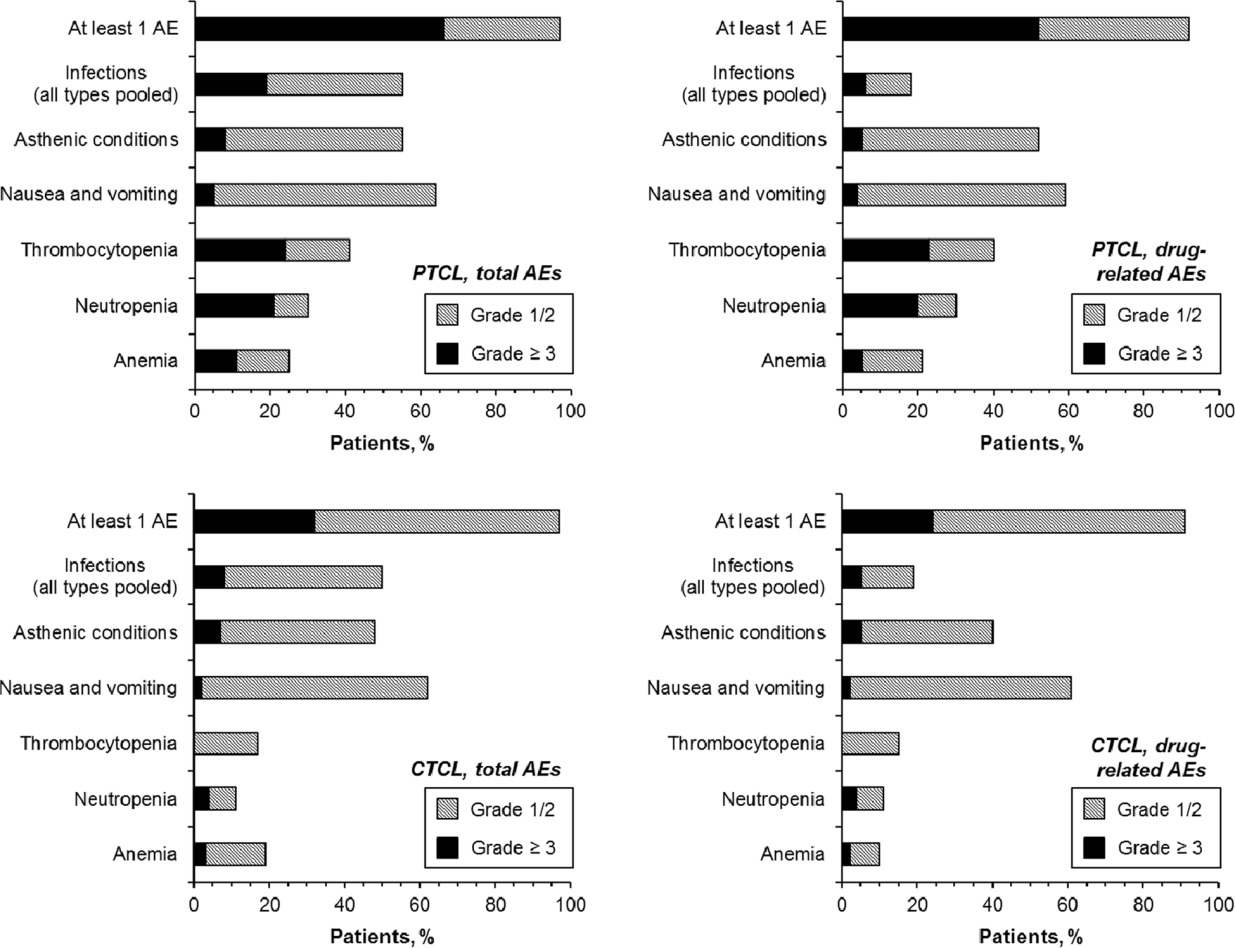
Total adverse events (AEs) and drug-related AEs in patients with relapsed/refractory peripheral or cutaneous T-cell lymphoma (PTCL, CTCL).

**Figure 2:**
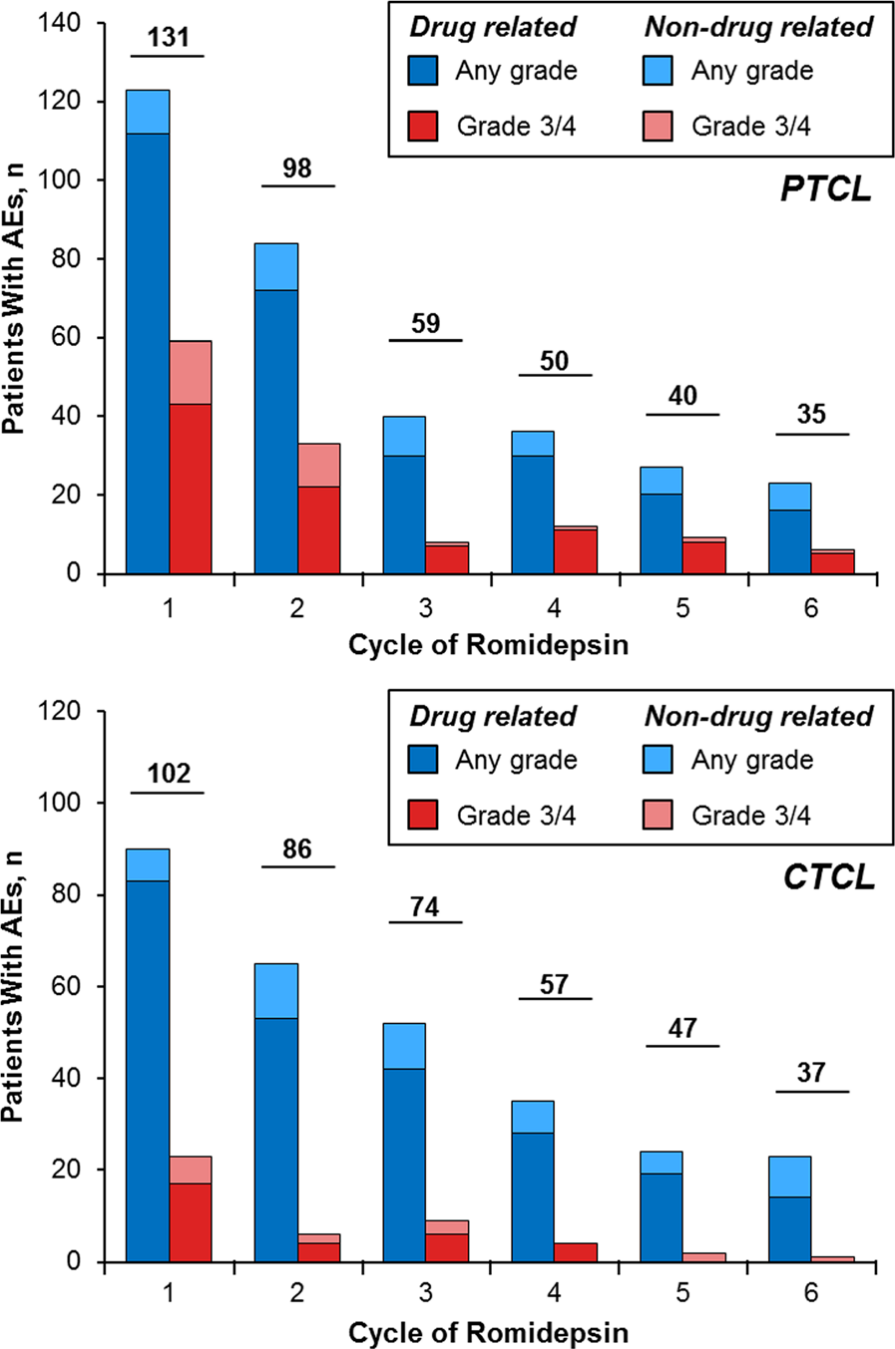
**Incidence of any grade and grade ≥ 3 adverse events (AEs) by cycle for patients with relapsed/refractory peripheral or cutaneous T-cell lymphoma (PTCL, CTCL).** Numbered bars represent number of patients treated in each cycle.

A similar safety profile of common AEs was seen in the PTCL and CTCL populations, and nausea/vomiting was the most frequent AE and drug-related AE (all grade) in both populations (Figure 1). Overall, a numerically higher incidence of grade ≥ 3 AEs (66% and 52% vs 32% and 24% for total or drug-related grade ≥ 3 AEs for patients with R/R PTCL or CTCL, respectively) and a higher incidence of hematologic toxicities (24%, 21%, and 11% grade ≥ 3 thrombocytopenia, neutropenia, and anemia, respectively, for patients with R/R PTCL and 0%, 4%, and 3%, respectively, for patients with R/R CTCL) were reported for patients with R/R PTCL. The incidences of grade ≥ 3 infections (all types pooled) were 19% and 8% for patients with R/R PTCL or CTCL, respectively, although the majority of infections were not related to romidepsin treatment per the investigator (6% and 5% drug-related grade ≥ 3 infections for patients with R/R PTCL or CTCL, respectively; Figure 1, Table 1). In both the R/R PTCL and CTCL populations, the incidence of all grade and grade ≥ 3 AEs (overall and drug-related) was highest during the first few cycles of treatment (Figure 2) and declined thereafter.

**Table 1:** Listings of infections with overall incidence in > 5% of patients with PTCL or CTCL

	**Total AEs**		**Drug-related AEs**	
**Subgroup**	**All grade**	**grade ≥ 3**	**All grade**	**Grade ≥ 3**
Cellulitis, n (%)				
PTCL	6 (5)	5 (4)	3 (2)	3 (2)
CTCL	2 (2)	0	0	0
Pneumonia, n (%)				
PTCL	8 (6)	6 (5)	2 (2)	2 (2)
CTCL	1 (0)	0	0	0
Sepsis, n (%)				
PTCL	7 (5)	7 (5)	2 (2)	2 (2)
CTCL	4 (4)	4 (4)	2 (2)	2 (2)
Nasopharyngitis, n (%)				
PTCL	6 (5)	0	0	0
CTCL	4 (4)	0	0	0
Upper respiratory tract infection, n (%)				
PTCL	11 (8)	2 (2)	7 (5)	2 (2)
CTCL	6 (6)	0	1 (1)	0
Urinary tract infection, n (%)				
PTCL	9 (7)	1 (1)	2 (2)	1 (1)
CTCL	3 (3)	0	1 (1)	0
Skin infection, n (%)				
PTCL	2 (2)	0	1 (1)	0
CTCL	7 (7)	1 (1)	2 (2)	0

AE, adverse event; CTCL, cutaneous T-cell lymphoma; PTCL, peripheral T-cell lymphoma.

### Impact of patient characteristics on toxicity profile in patients with R/R PTCL

**Figure 3:**
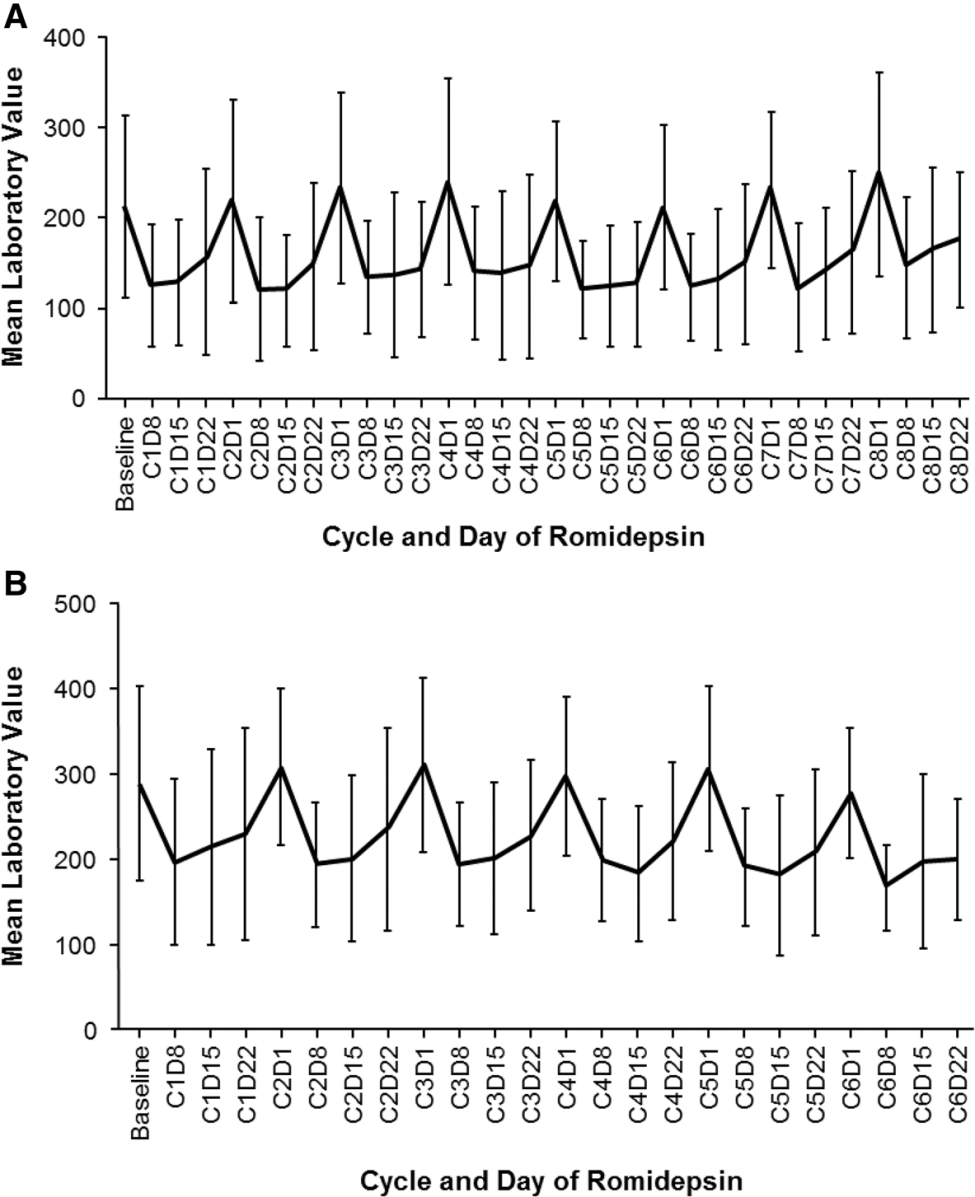
**Thrombocytopenia recovery by cycle of romidepsin treatment for patients with relapsed/refractory peripheral T-cell lymphoma (A) or cutaneous (B) T-cell lymphoma.** BL, baseline; C, cycle, D, day.

Common AEs were examined by subgroups of patients with R/R PTCL to determine whether baseline or disease characteristics can predict tolerability to romidepsin. The factors considered were PTCL subtype, age, International Prognostic Index score, type and number of prior therapies, and presence of bone marrow involvement. The incidence of treatment-related grade ≥ 3 infection (20% vs 4%) and neutropenia (50% vs 14%) was higher in patients with R/R PTCL who received prior monoclonal antibody (MAb) therapy (primarily alemtuzumab [n = 7] or rituximab [n = 11]); likewise there was a higher incidence of treatment-related grade ≥ 3 thrombocytopenia (38% vs 15%) (Table 2) in the more heavily pretreated patients. While patients who had received prior MAbs had lower baseline blood counts compared to other patients in the studies, there was no relationship between baseline lab values and incidence of grade ≥ 3 cytopenias. For the majority of patients with PTCL or CTCL, platelet count recovered between each treatment cycle, consistent with the reported mechanism for romidepsin-induced thrombocytopenia (Figure 3) [21]. The incidence of treatment-related grade ≥ 3 AEs was not high enough to perform an equivalent analysis for patients with R/R CTCL, and only 4 CTCL patients had prior MAb exposure.

**Table 2:** **Incidence of treatment-related grade ≥ 3 adverse events by patient characteristics in patients with PTCL**^**a**^

	**No.**	**Infection**	**Thrombocytopenia**	**Neutropenia**	**Anemia**	**Asthenic conditions**	**Nausea and vomiting**
PTCL subtypes							
PTCL NOS	69	4	20	17	4	7	4
AITL	27	4	30	22	7	4	0
ALK-1–negative ALCL	21	5	24	14	0	5	5
Other	14	21	31	36	14	0	7
Age							
< 65 years	86	6	23	22	6	4	6
≥ 65 years	45	7	22	16	4	9	0
International Prognostic Index score							
0-1	31	3	19	13	10	0	7
≥ 2	100	7	24	22	4	7	3
Prior systemic therapies							
< 3	83	6	15^b^	21	8	5	5
≥ 3	48	6	38^b^	19	0	6	2
Prior stem cell transplant							
Yes	21	0	33	10	5	5	0
No	110	7	21	22	6	6	5
Prior monoclonal antibody therapy^c^							
Yes	20	20^d^	35	50^e^	5	5	0
No	111	4^d^	21	14^e^	5	5	5
Bone marrow involvement							
Yes	37	8	30	24	5	5	0
No	94	5	20	18	5	5	5

AITL, angioimmunoblastic T-cell lymphoma; ALCL, anaplastic large cell lymphoma; ALK, anaplastic lymphoma kinase; PTCL NOS, peripheral T-cell lymphoma not otherwise specified.

^a^All comparisons except those noted below were not significant (*P* > .05).

^b^Primarily rituximab or alemtuzumab.

^c^*P* = .005.

^d^*P* = .019.

^e^*P* < .001.

### Serious AEs, hospitalizations, dose adjustments, and discontinuations

**Figure 4:**
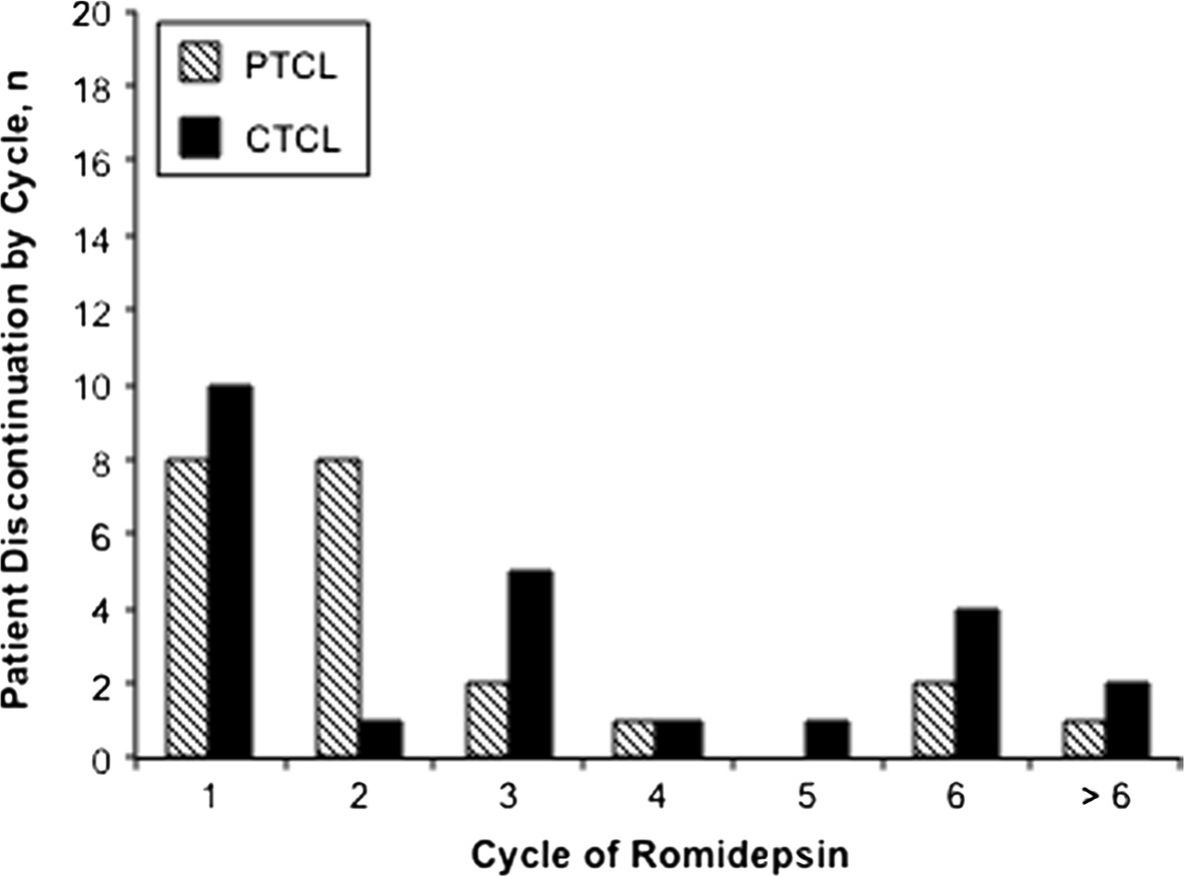
Patient discontinuation due to adverse events by cycle of romidepsin treatment in patients with relapsed/refractory peripheral or cutaneous T-cell lymphoma (PTCL, CTCL).

Infections were the most common serious AEs and reason for hospitalization in patients with R/R PTCL or CTCL (Table 3). Dose interruptions due to AEs occurred in 47% of patients with R/R PTCL and 34% of patients with R/R CTCL. Dose reductions due to AEs occurred in 11% of patients with R/R PTCL and 14% of patients with R/R CTCL (Table 4). For patients with PTCL, dose interruptions were most commonly due to thrombocytopenia (18%), infections (all types pooled; 12%), and neutropenia (11%); thrombocytopenia was the only AE that led to dose reduction in > 2 patients (n = 4). For patients with CTCL, dose interruptions were most commonly due to infections (all types pooled; 13%), neutropenia (3%), fatigue (3%), and hypomagnesemia (3%); vomiting was the only AE that led to dose reduction in > 2 patients (n = 3). Rates of discontinuation due to AEs were low in both patient populations (Table 3). The majority of discontinuations due to AEs for patients with R/R PTCL occurred during cycles 1–2 (Figure 4). For patients with R/R CTCL, 42% (10/24) of discontinuations occurred during cycle 1 (Figure 4).

**Table 3:** Total and drug-related serious adverse events, hospitalizations, and discontinuations

	**Total AEs**			**Drug-related AEs**		
**Subgroup**	**SAEs**	**Hosp**	**D/C**	**SAEs**	**Hosp**	**D/C**
Anemia, n (%)						
PTCL	2 (2)	2 (2)	0	2 (2)	2 (2)	0
CTCL	1 (1)	1 (1)	0	1 (1)	1 (1)	0
Neutropenia, n (%)						
PTCL	7 (5)	7 (5)	2 (2)	5 (4)	5 (4)	2 (2)
CTCL	2 (2)	0	1 (1)	2 (2)	0	1 (1)
Thrombocytopenia, n (%)						
PTCL	2 (2)	1 (1)	3 (2)	1 (1)	1 (1)	3 (2)
CTCL	0	0	1 (1)	0	0	1 (1)
Nausea and vomiting, n (%)						
PTCL	6 (5)	6 (5)	0	4 (3)	4 (3)	0
CTCL	0	0	0	0	0	0
Asthenic conditions, n (%)						
PTCL	2 (2)	2 (2)	2 (2)	0	0	1 (1)
CTCL	2 (2)	1 (1)	4 (4)	1 (1)	1 (1)	4 (4)
Infections, n (%)						
PTCL	25 (19)	21 (16)	6 (5)	6 (5)	3 (2)	2 (2)
CTCL	8 (8)	8 (8)	5 (5)	5 (5)	5 (5)	3 (3)

AE, adverse event; CTCL, cutaneous T-cell lymphoma; D/C, discontinuations; Hosp, hospitalizations; PTCL, peripheral T-cell lymphoma; SAEs, serious adverse events.

**Table 4:** Dose reductions and discontinuations

**Characteristic**	**PTCL (N = 131)**	**CTCL (N = 102)**
Dose reduction due to adverse events, n (%)	14 (11)	14 (14)
Dose interruptions due to adverse events, n (%)	61 (47)	35 (34)
Discontinuation, n (%)		
Progressive disease	78 (60)	22 (22)
Adverse event	22 (17)	24 (24)
Adverse event related to romidepsin treatment	11 (8)	17 (17)
Other^a^	7 (5)	26 (26)

CTCL, cutaneous T-cell lymphoma; PTCL, peripheral T-cell lymphoma.

^a^Most often refers to withdrawal of consent.

The most common AEs leading to discontinuation for patients with R/R PTCL were thrombocytopenia in 3 patients and infection in 6 patients (3 pneumonia, 2 sepsis, 1 upper respiratory infection) and for patients with R/R CTCL were asthenic conditions in 4 patients and infection in 5 patients (1 Epstein-Barr viral infection, 4 upper respiratory tract infections) (Table 3).

## Discussion

Single-agent romidepsin has demonstrated clinical activity in patients with R/R CTCL (34% ORR including 6% CR and median DOR of 15 months) [18] and PTCL (25% ORR including 15% confirmed/unconfirmed CR and median DOR of 28 months) [19]. The types of common AEs reported with romidepsin therapy were similar across the 2 indications, including hematologic toxicities, gastrointestinal events, asthenia/fatigue, or infections [13,16-18]. Approved romidepsin dosing was generally well tolerated in patients with R/R CTCL or PTCL, with AEs resulting in < 20% of patients discontinuing therapy and < 15% of patients requiring dose reductions.

Despite the similar AE profiles, there were some notable differences across the indications. Patients with R/R PTCL experienced more frequent and more severe hematologic toxicities and more frequent grade ≥ 3 infections (all types pooled). Although it is recognized that the cause of the thrombocytopenia with romidepsin is not due to a direct myelosuppressive effect but rather to defective megakaryocytic budding [21], it seems likely that the hematologic AEs observed in patients with PTCL may be attributed to a reduced megakaryocyte pool due to prior myelosuppressive chemotherapy and/or bone marrow disease involvement [13]. In this study, patients with R/R PTCL who had received ≥ 3 prior systemic therapies had significantly higher treatment-related grade ≥ 3 thrombocytopenia compared with those who had < 3 prior systemic therapies. Prior monoclonal antibody exposure in patients with R/R PTCL (predominantly alemtuzumab or rituximab) was associated with a significantly higher incidence of treatment-related grade ≥ 3 infection and neutropenia. This analysis could not be performed for patients with R/R CTCL, as rates of treatment-related grade ≥ 3 infections were too low to assess differences, and only 4 patients had prior MAb exposure.

For this analysis, all infection types were pooled to accurately reflect the total infection risk. Because AE reporting separates each infection type, the incidence of each subset may fall below the cutoff of those reported as “common” AEs, and infection risk may be overlooked. Additionally, comparing infection risk across agents is difficult when they are not pooled. In the pivotal study of pralatrexate for the treatment of R/R PTCL, only the specific infections seen in ≥ 10% of patients (upper respiratory tract infection and sinusitis) were reported [22]. In patients with R/R CTCL treated with vorinostat, only upper respiratory infection was reported in ≥ 10% of patients [23]. No specific infection type was reported in ≥ 10% of patients with R/R PTCL or CTCL treated with romidepsin. However, when pooled, 55% of patients with R/R PTCL and 50% of patients with R/R CTCL (19% and 8% grade ≥ 3, respectively) experienced infection during romidepsin treatment. Notably, most of the infections were not considered related to romidepsin treatment and thus may have been due to the increased risk of infection related to disease state or prior therapies. As a result of compromised skin integrity, patients with CTCL experience frequent infections [9,10,24,25].

Because of the risk of infection in patients with T-cell lymphoma, steps to minimize infectious complications should be taken. Romidepsin does not require a central IV catheter, which has been associated with an increased risk of infection in patients with CTCL [26]. Strict adherences to aseptic technique should be taken when establishing a peripheral IV line for drug administration. Additionally, for patients with poor immune function due to heavy pretreatment or prior monoclonal antibody therapy, prophylactic antibiotic or antiviral medication may be appropriate.

Electrocardiogram changes were described in studies with romidepsin [27-29]; however, there was no evidence of myocardial damage or significant changes in left ventricular ejection fraction in romidepsin clinical trials with routine cardiac monitoring [28,29]. It was shown that clinically insignificant QTc effects observed with romidepsin treatment were likely related to antiemetic administration [20,28,30]. Changes in ECG parameters, including QTc intervals, are a class effect of 5-hydroxytryptamine 3 receptor agonist antiemetics [31,32]. Granisetron may have less of an effect on the QT interval than other 5-hydroxytryptamine 3 receptor agonist antiemetics [32]. Despite the use of prophylactic antiemetics, > 50% of patients with R/R PTCL or CTCL treated with romidepsin experience drug-related nausea and/or vomiting, although the majority of events are grade 1–2 [4,29].

The greatest incidence of all grade and grade ≥ 3 AEs and the majority of discontinuations due to AEs occurred during cycles 1–2. Early discontinuations primarily related to infection, thrombocytopenia, or ECG abnormalities confirm the need to closely monitor heavily pretreated patients, in particular those who have received prior monoclonal antibody therapy or those with comorbidities, with consideration of prophylactic antibiotic or antiviral medication, or a lower initial romidepsin dose of 10 or 12 mg/m^2^ in such patients. Despite this, however, 28% and 36% of patients with R/R PTCL or CTCL, respectively, tolerated romidepsin for at least 6 cycles.

## Conclusions

Single-agent romidepsin leads to durable response in a subset of patients with R/R PTCL or CTCL. This study demonstrated that patients with R/R PTCL or CTCL have similar AE profiles with romidepsin treatment, although patients with PTCL experienced more frequent and more severe hematologic toxicities and more frequent grade ≥ 3 infections. Extended dosing of romidepsin is feasible in responding patients without cumulative toxicities.

## Methods

### Study design and eligibility criteria

Study design and eligibility criteria for these 2 similarly designed phase 2, non-randomized, international, multicenter, single-arm studies in R/R CTCL (GPI-04-0001) or R/R PTCL (GPI-06-0002) were previously described [13,18]. GPI-04-0001 was a study of romidepsin for the treatment of patients with stage IB to IVA CTCL who had received at least one prior systemic therapy [18]. GPI-06-0002 was a study of romidepsin for the treatment of patients with histopathologically confirmed PTCL who had received at least one prior systemic therapy.

Patients on both trials had adequate organ function and no known significant cardiac abnormalities (eg, congenital long QT syndrome, QTc interval > 480 msec, recent myocardial infarction, coronary artery disease, congestive heart failure, cardiac arrhythmia requiring anti-arrhythmic medications, ventricular tachycardia, ventricular fibrillation, torsades de pointes, or cardiac arrest). Concomitant use of drugs known to significantly prolong the QTc interval and CYP3A4 inhibitors was disallowed. Because hypokalemia and hypomagnesemia can be associated with electrocardiogram (ECG) abnormalities [28], patients must have had normal levels of serum potassium and serum magnesium; low levels could be corrected with supplementation to meet inclusion criteria. Both studies were conducted in accordance with the Guidelines of the World Medical Association Declaration of Helsinki in its revised edition (Washington, 2002), the guidelines for current Good Clinical Practice (CPMP/ICH/135/95), the requirements of Directive 2001/20/EC (European Investigators only), all applicable US FDA regulations (US Investigators only) as well as the demands of national drug and data protection laws and other applicable regulatory requirements, as appropriate. For both trials, the protocol, informed consent form, and other relevant study documentation were approved by the appropriate institutional review board or independent ethics committee at each study site (for primary author: Yale University School of Medicine Human Investigation Committee, Suite 204, 47 College St, New Haven, CT 06520). All patients provided written informed consent before any study-specific procedure was performed.

Patients on both trials received romidepsin 14 mg/m^2^ as a 4-hour intravenous infusion on days 1, 8, and 15 of each 28-day cycle for up to 6 cycles; patients with at least stable disease could extend therapy until progressive disease or another withdrawal criterion was met. This dose and schedule were based on results from the NCI phase 2 trials of patients with R/R CTCL [16] or PTCL [17], and is the Food and Drug Administration–approved dosing in both indications [4].

### Safety assessments

Adverse event (AE) recording was similar in both the CTCL and PTCL studies, with toxicities classified according to the Medical Dictionary for Regulatory Activities Version 12.0, and AE severity was graded according to NCI Common Terminology Criteria for Adverse Events Version 3.0. AEs were recorded on days 1, 8, and 15 of every cycle for all patients; patients with R/R PTCL also had AEs recorded on day 22 of every cycle. Drug-related AEs were defined as those considered by the investigator to have a possible, probable, or very likely/certain relationship to the study drug. Dose adjustment criteria were similar across the 2 studies. Briefly, grade 3–4 nonhematologic toxicities and grade 3–4 neutropenia or thrombocytopenia resulted in dose interruption. One permanent dose reduction to 10 mg/m^2^ romidepsin as a 4-hour IV infusion on days 1, 8, and 15 of each 28-day cycle was permitted. Following dose reduction, if the AE recurred, the patient was discontinued from the study. Common AEs reported during these studies, specifically anemia, neutropenia, thrombocytopenia, nausea/vomiting, asthenic conditions, and infections (all types pooled), are the focus of the analyses described in this manuscript.

### Statistical methods

In both studies, all enrolled patients who received at least one dose of romidepsin were included in the safety analysis. In the analysis of treatment-related grade ≥ 3 AEs by patient characteristics, the percentages of patients with common AEs in various patient subgroups were compared by Fisher’s exact test, with *P* < .05 demonstrating significant differences in AE rates.

## Competing interests

FF; Advisory Board, Consultant: Celgene Corporation, Spectrum Pharmaceuticals, Seattle Genetics, Millennium Pharmaceuticals, Allos Pharmaceuticals; Clinical Trial Support: Celgene Corporation, Allos Pharmaceuticals, Eisai; Consultant: Eisai. BC; Advisory Board: Celgene Corporation. SH; Grant: Celgene Corporation, Millennium Pharmaceuticals, Infinity, Kyowa-Kinn, Seattle Genetics, Spectrum Pharmaceuticals, Consulting and Honoraria: Celgene Corporation, Millennium Pharmaceuticals, Amgen, Inc., Bristol-Myers Squibb Company, Janssen Pharmaceuticals. BP; Honoraria: Celgene Corporation. HMP; Research Grant and Honoraria: Celgene Corporation. LS; Clinical Trial Financial Support, Advisory Board, Consultation: Celgene Corporation. JN; Former Employee: Celgene Corporation. BB, JW; Employer received payment for manuscript statistical assistance: Veristat, Inc. MG, AL, DC, EB, EK, SW; None.

## Authors’ contributions

FF interpreted the data, drafted the paper, and approved all versions including the final version. BC, SH, BP, HMP, LS, MG, AL, DC, EB, EK, JN, SW interpreted the data, critically revised the paper, and approved all versions including the final version. BB and JW acquired and analyzed the data, critically revised the paper, and approved all versions including the final version.
